# When is RCH not RCH? Rapid cold hardening has steep temperature thresholds inducing high survival but low fertility resilience to cold stress

**DOI:** 10.1242/jeb.250856

**Published:** 2026-01-30

**Authors:** Jasmine R. Vidrio, Daniel A. Hahn, Michael P. Moore, Gregory J. Ragland

**Affiliations:** ^1^ Department of Integrative Biology, University of Colorado Denver, 1151 Arapahoe St, Denver, CO 80204, USA; ^2^ Department of Entomology and Nematology, University of Florida, Gainesville, FL 32611, USA

**Keywords:** *Drosophila melanogaster*, Acclimation, Plasticity, Hormesis

## Abstract

Variable thermal environments may have both detrimental and beneficial effects. For example, extreme temperatures may challenge homeostasis and inflict tissue damage, but may also induce acclimation that improves stress resilience. Hormetic models provide a framework to understand dosage-dependent, contrasting beneficial and detrimental effects from physiological and ecological perspectives. We used a hormetic framework and associated quantitative models to investigate how a range of relatively cold, pre-exposure temperatures influence survival and fertility following cold shock at a more extreme cold temperature in the fruit fly *Drosophila melanogaster*. Cold pre-exposure can induce a protective rapid cold hardening (RCH) response, fail to stimulate a response, or cause direct cold injury. We found a plateau-shaped relationship between pre-exposure temperature and female survival resilience, where survival following a cold shock remained high across a range of temperatures, with sharp transitions at higher and lower temperatures. Bayesian fitting of a bi-logistic model highlights these transitions at temperature thresholds that govern processes mediating both acclimation and cold injury. In contrast to survival, female fertility resilience exhibited a muted response to pre-exposure temperature in the presence and absence of post-stress mating opportunities. Overall, a range of pre-exposure temperatures allowed low but successful reproduction following cold shock. High survival but low fertility resilience is consistent with (1) differential impacts of cold on somatic and reproductive tissues and (2) a growing body of literature suggesting that the thermal sensitivity of fertility may be more limiting than survival for population persistence in variable and changing climates.

## Teaser

**Summary:** A hormetic model shows how sharp temperature thresholds govern beneficial rapid cold hardening and detrimental cold injury that have well-defined effects on survival, but only weakly affect fertility.

## INTRODUCTION

Variable thermal environments pose a formidable challenge to life, especially when temperatures surpass physiological thresholds for the loss of homeostasis or for induction of cold and heat damage (Schulte, 2014; Williams et al., 2015; Ørsted et al., 2022). In contrast, variable temperatures also provide opportunities for acclimation, or adjustment of physiology to maintain homeostasis or defend against damage in response to potentially damaging temperatures (Morley et al., 2019; Teets et al., 2023). The net effect of fluctuating temperatures on fitness will depend on the range of temperatures and the duration of exposure, which in turn influence both beneficial processes such as increased cryoprotectant concentrations in preparation for low temperatures (Bale, 1987; Thomashow, 1999), and determinantal or pathological processes such as loss of homeostasis and accumulation of cellular damage (MacMillan et al., 2015a; Somero, 2020). Many studies have explored these beneficial or detrimental effects across ranges of temperatures. However, few studies have investigated how the contrasting effects of these two sets of processes may influence fitness outcomes over temperature ranges that may induce both beneficial and detrimental effects (Yordanov et al., 1986; Williams et al., 2012; Enzor and Place, 2014).

Particularly in ectotherms, nearly all aspects of physiology are plastic as a function of temperature. From an evolutionary perspective, plastic changes can increase, decrease or have no effect on lifetime reproductive output, i.e. fitness (Ghalambor et al., 2007). In general, plasticity that increases or decreases fitness is termed adaptive or maladaptive plasticity, respectively. However, most studies only measure one or a few components of fitness, and the same temperature conditions may enhance one fitness component, while negatively impacting others (Kristensen et al., 2008). Thus, whether plasticity is adaptive is often inferred only in the context of one or a few measured traits over some pre-defined, experimental range of environments. Though these complexities mean that strong inferences about adaptive plasticity are difficult to achieve, the general framework of defining physiological impacts of plasticity with respect to fitness is generally useful (e.g. Schulte, 2014). Here, we avoid the word ‘adaptive’ and consider only whether a plastic response is beneficial or detrimental with respect to specific fitness components.

The combined effects of pathological and protective processes may often determine whether plastic responses to temperature are beneficial or detrimental. For example, a brief exposure to sub-lethal, high temperature may cause protein misfolding, negatively impacting multiple cellular and higher-level physiological processes (Hofmann and Somero, 1995). However, that same exposure may induce the production of chaperone proteins such as heat shock proteins (Hsps) that can mitigate protein misfolding during subsequent exposure to even more extreme high temperatures (Sørensen et al., 2007). Indeed, sub-lethal, short-term exposures to high temperature can simultaneously damage cellular components and also stimulate the classic heat shock response that can be highly beneficial in variable environments (Klepsatel et al., 2016).

Hormesis, a biphasic response to an environmental factor, provides a suitable conceptual and quantitative framework to model these contrasting, beneficial versus detrimental temperature effects. Frequently applied in toxicology, hormesis describes a non-monotonic, biphasic dose–response curve, where a ‘dose’ of some factor (e.g. toxin, radiation, temperature, etc.) increases some measure of performance at low doses, but decreases performance at higher doses (Calabrese and Baldwin, 2001). Beyond toxicology, hormetic models can be valuable in understanding how organisms respond to environmental stressors and can offer insights into ecological processes (Costantini et al., 2010; Berry and López-Martínez, 2020).

**Fig. 1.**
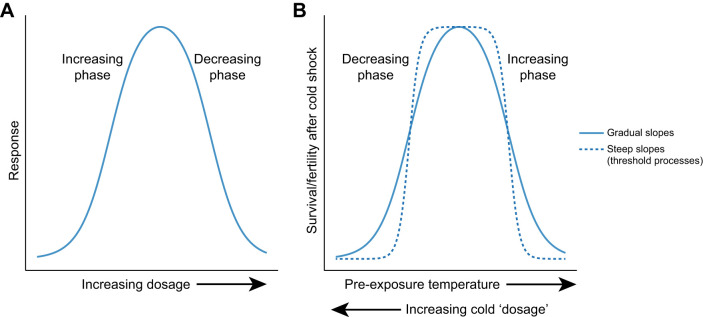
**Conceptual diagrams of hormetic models.** (A) Generic (hypothetical) model depicting a biphasic relationship where some measured response initially increases as a function of increasing dosage (increasing phase) but eventually decreases at higher doses (decreasing phase). (B) Hormetic (hypothetical) models relating cold pre-exposure to survival or fertility following a subsequent cold shock. Here, colder pre-exposures represent higher dosages, with initial decreases in temperature increasing survival/fertility after cold shock (increasing phase), and colder pre-exposures eventually decreasing survival/fertility (decreasing phase) after cold shock. The solid line in B models gradual slopes, whereas the dashed line models a relationship wherein processes with tight temperature thresholds underlie the observed response.

Here, we developed and applied a biphasic model to understand the relationship between the intensity of an initial cold exposure and components of fitness measured following subsequent exposure to even more extreme cold in the fruit fly *Drosophila melanogaster* (Fig. 1). Under certain conditions, exposing insects to moderate duration (often 1–4 h) and moderate intensity (often ∼0°C) cold vastly improves survival of a subsequent, more extreme (often ∼1 h at ≤−5°C) cold stress. This short-term acclimation response is known as rapid cold hardening (RCH) and has been well studied as a putative example of adaptive plasticity (or ‘beneficial acclimation’; Woods and Harrison, 2002) and as a model for rapid physiological adjustments to the environment (Teets et al., 2020). RCH appears to be a widespread phenomenon in arthropods that has also been observed in vertebrates (Teets et al., 2020). Although RCH has often been assessed with respect to survival after extreme cold exposure, RCH also affects other fitness components, e.g. dispersal and reproduction (Lee and Denlinger, 2010; Teets et al., 2020). The initial description of RCH was notable because of the rapidity of acclimation, but also because of the astonishingly strong effects – RCH yielded greater than two-fold increases in survival of extreme cold across multiple insect species, and in some cases increased survival from 0% (no cold pre-treatment) to >88% with RCH (Lee et al., 1987). These observations imply a positive relationship between survival and intensity of cold pre-treatment up to a point, conforming to the increasing phase of a hormetic curve where ‘dose’ is the temperature (colder is a higher dose; Fig. 1B). But as the pre-treatment temperature approaches the extreme cold temperature applied in the post-treatment, survival must eventually decline, conforming to the decreasing phase of the hormetic curve. Though the general form of this relationship is self-evident (at least for survival), to our knowledge there are no studies that explicitly model the full function.

We performed experiments across a broad range of pre-treatment temperatures to model hormetic curves relating pre-exposure temperature to survival and fertility following extreme cold stress in *D. melanogaster*. This experimental design allowed us to model hormesis in the framework of the standard design for RCH experiments. We also identified an RCH study applying a range cold pre-exposure treatments followed by cold shock to pharate adults of the flesh fly, *Sarcophaga crassipalpis* (Chen et al., 1987). We fit a biphasic model to the results of that study as well to provide a comparison of model suitability and hormetic curve shape across fly species.

Beyond illustrating the utility of biphasic models for understanding both beneficial and detrimental effects of thermal exposures, we had two primary goals.

First, we sought to draw inferences about mechanisms underlying beneficial and detrimental effects of cold exposure. Specifically, we asked whether RCH and cold-damage effects accumulate gradually with temperature, or whether there are steep thresholds for induction. Here, we define ‘gradual’ as the expected thermal sensitivity of enzymatic or metabolic functions often modeled by the Arrhenius equation (Clarke and Fraser, 2004; Crapse et al., 2021). The relationship between temperature and rate (e.g. development rate or metabolic rate) is commonly expressed as a *Q*_10_ coefficient (the change in rate across 10°C temperature change). Development and metabolic rates generally have *Q*_10_ coefficients of approximately 2–5, corresponding with the typical *Q*_10_ coefficients for chemical reactions and metabolic processes (Dell et al., 2011; Maynard Smith, 1957). In contrast, the relationship between survival times at stressful high temperatures may be relatively steep, with *Q*_10_ coefficients 2–4 orders of magnitude higher (Maynard Smith, 1957; Jørgensen et al., 2022). If the physiological effects of temperature accumulate gradually as expected for enzymatic or metabolic reactions, we would expect shallow slopes with *Q*_10_ coefficients of approximately 2–5 relating pre-treatment temperature to fitness components and a relatively narrow range of temperatures over which the fitness component is near the maximum (e.g. Fig. 1B, solid line). However, if sharp temperature thresholds underlie the induction, then cessation of survival resilience at increasingly colder pre-exposure temperatures, we expect a relatively broad plateau at the maximum set off by steep slopes on either side with *Q*_10_ coefficients substantially greater than 5 (e.g. Fig. 1B, dashed line). Note that *Q*_10_ as usually calculated is less than 1 in the increasing phase (where decreasing temperatures increase resilience), in which case the prediction is an inverse of *Q*_10_ greater than 5. Here, we assume that the measured fitness component is the cumulative result of potentially multiple processes with some average rate varying with temperature, e.g. proportion survival as a measure of rate of survival over some duration of exposure.

Second, we sought to compare the thermal sensitivity of two fitness components, survival and fertility, as a function of pre-treatment temperature. Several aspects of reproduction often demonstrate distinct thermal sensitivity compared with survival and other thermal performance metrics (Walsh et al., 2019). Moreover, survival cannot improve fitness unless successful reproduction follows.

### A note on language and terminology associated with the study of RCH plasticity

We have found that the dual cold exposures, the contingency of measured responses on at least one stressful treatment, and the expectation of a beneficial effect in the name (‘hardening’) can lead to relatively convoluted and sometimes biased language describing RCH experimental results. Below, we simplify the language where possible in three ways. First, we use the term ‘resilience’ to describe the degree of preservation of fitness components (here, survival and fertility) following exposure to a severe stressor (here, severe cold shock). This avoids the need to repeatedly frame the response (e.g. survival) in the context of stressor exposure (e.g. survival following cold shock). Second, we refer to the first temperature exposure treatment simply as a ‘pre-exposure’, which then may or may not improve resilience relative to other pre-exposure conditions. Third, we use the term harden or hardening to specifically describe an outcome in which pre-exposure temperature improves resilience relative to control treatments that do not apply a pre-exposure colder than standard rearing temperature. Though we do not apply a mathematical definition of resilience in this study, we include a brief discussion proposing a mathematical definition that may be practical and improve clarity for some experimental designs in our Discussion.

## MATERIALS AND METHODS

### Fly lines and husbandry

We performed all experiments on female flies derived from stocks of the isogenic Oregon R line of *Drosophila melanogaster* Meigen 1830 reared in the lab at the University of Colorado, Denver, for approximately 5 years. We used only mated female flies because: (1) male and female flies differ in thermal tolerance (Bubliy et al., 2002), and (2) we could easily assay female fertility based on offspring production. We reared all experimental flies in vials on a standard cornmeal–agar–molasses diet (see Table S1 for recipe) in a Percival DR-36VL incubator (Percival Scientific, Perry, IA, USA) set to 24°C and a 14 h:10 h light:dark cycle.

### Survival experiments

We initially determined that directly exposing flies reared at 24°C to −7°C for 1 h led to 100% mortality, whereas pre-exposure to a colder temperature frequently used in other RCH studies (4°C) for 210 min (as in Gerken et al., 2018) led to intermediate mortality following cold shock (30–35%). Using −7°C as the cold-shock temperature therefore allowed us to assess both increases and decreases in mortality with pre-exposures above and/or below 4°C. We further note that *D. melanogaster* adults supercool to temperatures below −16°C (Czajka and Lee, 1990), so we do not expect that the flies experienced internal ice formation either during our 210-min pre-exposures that ranged from −1°C to 14°C or during our 60-min stress exposure at −7°C. All experiments were performed on flies that were (1) anesthetized under CO_2_ in order to sort flies by sex into new vials with food 3–4 days post-eclosion, (2) allowed 24 h to recover from anesthesia, and then (3) tap-transferred into empty vials. Using the direct transfer RCH method from Gerken et al. (2018), we transferred fly vials from the rearing temperature of 24°C directly into an Arctic A40 recirculating chiller (Thermo Fisher Scientific, Waltham, MA, USA) containing 50% (v/v) propylene glycol that was set at the desired pre-exposure temperature. Vials were held at the pre-exposure temperature for 210 min, then the bath temperature was dropped at a constant rate over 5 min to the acute cold-shock temperature of −7°C, then held there for 60 min. To monitor the bath temperature, we placed two Type T thermocouples (Omega Engineering, Norwalk, CT, USA) interfaced with PicoLog v6 via TC-08 units (Pico Technology, Cambridgeshire, UK) into each of two empty vials (four thermocouples total) placed proximal to vials containing experimental flies. Vial temperatures were always within 0.22°C of the intended pre-treatment or cold-shock temperature (see data archive on figshare at https://doi.org/10.6084/m9.figshare.31100800.v1). Following the cold shock, flies were gently tapped into new food-containing vials and immediately transferred back to 24°C. Vials were placed onto their sides during transfers and for 24 h following transfers to avoid comatose flies sticking to food. After 24 h, flies were scored as ‘live’ if they were able to stand on their legs (David et al., 1998).

**Fig. 2.**
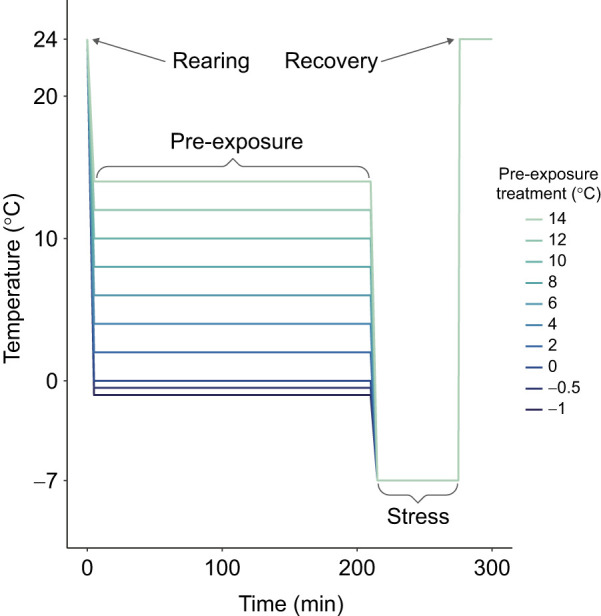
**Conceptual illustration of experimental treatments.** Each treatment (line) used common rearing, cold-shock and recovery temperatures, with only pre-exposure temperature varying among treatments.

We applied a broad range of pre-exposure temperatures, attempting to identify a range of temperatures causing high survival resilience, and temperatures above and below that range causing relatively low survival resilience. In initial pilot experiments, we also noted a rapid decline in survival with pre-exposure temperatures below 0°C and thus applied more closely spaced pre-exposure temperatures at the low end of the pre-exposure temperature range. We applied the following pre-exposure treatments: 210 min at −1, −0.5, 0, 2, 4, 6, 8, 10 and 14°C. Fig. 2 illustrates the thermal profiles for each treatment – treatments varied in pre-exposure temperature but all included a common 60-min exposure to a −7°C cold shock. Each treatment included three to four replicate vials (see data archive on figshare at https://doi.org/10.6084/m9.figshare.31100800.v1), with each vial containing 20 females.

### Fertility assays

Following the survival assays described above, we tracked offspring production of surviving females. Counting eggs is a common method to assess fertility, but eggs may or may not be viable, and offspring may or may not successfully develop. We instead counted the number of pupae produced in each vial as in Hafezi et al. (2020). Females (without any males added) were allowed a total of 48 h following the cold-shock exposure to oviposit and then were cleared from the vials. We also created control vials containing four to ten 3-to-4-day-old females from the same cohort of flies used to initiate the experiments. Control flies were handled the same way as pre-exposure/cold-shock treated flies but were simply held at the rearing temperature (24°C) for the duration of treatment period. Recall that we used mated females that can continue to produce viable offspring from eggs fertilized prior to the experiments or using stored sperm. We counted total pupae produced from each vial up to 14 days following the experiment – no new pupae were ever observed past 14 days, much longer than the usual egg-to-pupa development time for *D. melanogaster* at 24°C (Ashburner et al., 2005).

After initially observing very low fertility across all pre-exposure treatments (see Results), we performed one additional experiment to test whether introducing untreated males to females after they had been cold shocked might improve fertility resilience. We repeated the assays as described above applying two pre-exposure temperatures, one where survival was relatively high (10°C pre-exposure) and one where survival was relatively low (14°C pre-exposure). We also included untreated control vials, as above. We modified the assay by splitting vials into two groups: one group had no males added (equivalent to the treatments described above), and one group had 20 untreated, non-virgin, 3–4 day post-eclosion males added per vial immediately following the return to 24°C following cold shock. Vials were cleared at 48 h, and pupae were counted as described above. Each pre-exposure and cold-shock treatment included four vials with and four vials without males, whereas each control treatment included one vial. If fertility in this experiment recovered after untreated males are added to the vials, the result would suggest that pre-exposure and/or cold shock is disrupting reproduction through its effects on the male contributions stored by females prior to their cold shock, perhaps through reduced sperm motility or viability. By contrast, if fertility did not recover after introducing untreated males to cold-shocked females, the result would suggest that pre-exposure and/or cold shock disrupts other aspects of female reproduction through, e.g. damage to somatic or germline reproductive tissues.

### Statistical analyses

Hormetic processes can be modeled using a bi-logistic function including both increasing and decreasing logistic phases (Beckon et al., 2008). Such functions can include easily interpretable parameters that describe, for example, the rate of increase/decrease, thresholds or positions of phases (often inflection points), and maximum response values (e.g. survival or fertility in this study). Initial inspection of the data suggested a non-monotonic, plateau-shaped function with asymmetric rates of increase and decrease. We designed a modified logistic, five-parameter model to incorporate these shape elements. Our model is a simplified variant of the biphasic model described in Beckon et al. (2008). This simplified model is less flexible in shape, but it provides a good fit to our survival data (see Results) and has the advantage of more straightforward parameter interpretation. We modeled the relationship as follows:
(1)

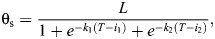
where θ_s_ is the proportion survival, *T* is the temperature, *L* models the upper limit of survival, *k*_1_ and *k*_2_ model the steepness of logistic change in the decreasing and increasing phases, respectively (Fig. 1B), and *i*_1_ and *i*_2_ model the temperatures of the two inflection points (the temperature at which the logistic changes in each phase are centered). Note that the codomain of the function is any real number; thus, it can be used to model data in different ranges (e.g. [0,1] for survival proportion) by constraining *L*. We fit the model using a Bayesian procedure implemented in Stan (6.1 Stan User's Guide Version 2.34) and accessed through the *cmdstanr* package in R version 4.3.1 (https://www.r-project.org/). We used the default No U-Turn Sampler (NUTS), a Hamiltonian Monte Carlo (HMC) method to sample the posterior distribution, running four sampling chains, each with a burn-in of 2000 iterations followed by 3000 retained samples per chain, for 12,000 samples total. Because survival proportion in some treatments was well above 0.5, we constrained *L* to [0.5,1] and we placed additional constraints and priors on some parameters based on visual inspection of the data to prevent non-convergence (see relevant scripts in the supplied data/code archive at https://doi.org/10.6084/m9.figshare.31100800.v1 and Supplementary Materials and Methods and Fig. S1 for details and rationale). We specified a binomial (*n*=number of total females in a vial, *k*=number of surviving females) likelihood function for θ_s_. All chains achieved stationarity with symmetrical, unimodal posterior distributions for each parameter. Finally, to visualize the modeled relationship with uncertainty, we calculated mean θ_s_ and the 95% credible interval of the posterior distribution for temperatures ranging from −3°C to 18°C at 0.01°C increments.

To assess whether the model fit well and predicted a similar relationship for a comparable set of data from another insect species, we fit the same bi-logistic function to estimates of the survival resilience to cold shock across pre-exposure temperatures reported in a separate study of *S. crassipalpis* pharate adults (Chen et al., 1987). We extracted estimated mean proportion survival versus pre-exposure temperature from fig. 3 in Chen et al. (1987) using PlotDigitizer (https://plotdigitizer.com/app). Though Chen et al. (1987) applied pre-exposure temperatures up to 35°C and noted a hardening response at high in addition to low temperature, we excluded temperatures above 25°C from our re-analysis because (1) such high temperatures are highly unlikely to immediately precede exposure to sub-zero temperatures in nature, and (2) our goal was to compare survival resilience over a comparable range of temperatures across the two datasets. We set the binomial parameter *n* to 60 for each trial (the study methods indicate 15–20 pupae in each of three replicate groups) and we set *k* to produce the estimated proportion survival reported in Chen et al. (1987). We did not estimate uncertainty (the 95% credible interval) for the model using the data from Chen et al. (1987) because we lacked raw data including survival rates per replicate group and exact sample sizes.

As discussed in the Introduction, one goal of the present study was to assess the steepness of the temperature–fitness component relationship as quantified by *Q*_10_ coefficients relative to expected *Q*_10_ values for enzymatic and metabolic function (*Q*_10_∼2–5). Because the hormetic model fit best with the survival data and relatively poorly for fertility (see below), we limited this exploration to the survival data only. We first applied an arcsin square root transform to survival proportion to minimize the influence of compression around the limits of 0 and 1 (these tend to lead to extremely high *Q*_10_ estimates). We then calculated *Q*_10_ as:
(2)

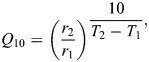
where *r*_2_ and *r*_1_ are the transformed survival proportions (rate of survival over the duration of the experiment) at temperatures *T*_2_ and *T*_1_. For the data from the present study and from Chen et al. (1987), we calculated the mean *Q*_10_ across three temperatures spanning the increasing phase and three temperatures spanning the decreasing phase (Fig. 1B).

During initial inspection of the survival resilience data, we noted that the shape of the relationship with our short-duration pre-exposure temperatures is very similar to that observed between insect egg to adult viability and long-term (lifetime) constant temperature exposure (van der Have, 2002). Thus, we generated estimates of *Q*_10_ for survival of long-term temperature exposures as an additional comparison. We again used PlotDigitizer to extract data relating adult survival to lifetime temperature exposure in three species of *Drosophila* flies (*D. melanogaster*, *D. simulans* and *D. yakuba*) from van der Have (2002). The results from fig. 9 in van der Have (2002) suggest a plateau-shaped relationship with very steep transitions at low (about 14°C) and high (about 30°C) temperature. We therefore used a similar approach, calculating mean *Q*_10_ across three temperatures spanning the decreasing phases of the relationships (temperatures between about 11°C and 14°C) to focus specifically on *Q*_10_ for mortality induced at lower temperatures.

Initial inspection of the first set of fertility data, including all pre-exposure temperatures from the survival experiment and without addition of males, suggested low fertility with high variance within temperature treatments. Attempted fitting of a modified version of the model described in Eqn 1 (using a negative binomial likelihood function) resulted in non-convergence with moderate constraints and priors comparable to the survival model above. We were able to fit the model with highly constrained parameter values and highly informative priors, which we present in the Supplementary Materials and Methods. However, the model fit was relatively poor and overly influenced by constraints and priors, and we provide the details in the Supplementary Materials and Methods only as a proof of concept that the model can be fit to different data distributions.

We chose to instead fit generalized linear models (GLMs) treating pre-exposure as a discrete factor to simply test whether fertility varied across pre-exposure temperature using the *glm* function in R. Though GLMs with Poisson and related error distributions require count data as the response, rates can be modeled by including an offset term (Dunn and Smyth, 2018). We used this approach to model the number of pupae per female using GLMs with total pupae per vial as the response, pre-exposure temperature as a discrete factor, and number of surviving females per vial as an offset. An initial fit using a Poisson model suggested overdispersed data using the simulation-based dispersion test from the *DHARMa* package in R. We settled on a negative binomial model with the same parameters and data, providing a good fit with no evidence of overdispersion. We then used the *predict* function to estimate mean number of pupae per female and associated 95% confidence intervals (CI) for each pre-exposure and control treatment.

The second fertility experiment included only two pre-exposure temperatures and a discrete factor for addition (or non-addition) of males. We thus applied a similar approach using negative binomial models with total pupae per vial as the response and number of surviving females per vial as an offset. We fit two models – both included a discrete factor for pre-exposure temperature and a discrete factor for male addition, but one also included an interaction. We used Akaike's information criterion (AIC) model selection to determine the best fit model, then used the *predict* function to estimate the mean number of pupae per female and associated 95% CI for each treatment combination.

Finally, we estimated replacement rate under laboratory conditions to illustrate how cold pre-exposures influence a composite metric of fitness including both survival and fertility after cold shock. For vials that were measured for both survival and fertility (all vials in the survival experiment that were also measured for fertility without the addition of males), we estimated laboratory replacement rate of the females as the product of survival proportion and fertility (pupae per female) estimated for each replicate vial. The variance in replacement rate within and among treatments appeared similar compared with estimated fertility. Thus, we did not attempt to fit a continuous, Bayesian model. Rather, we estimated means and approximate 95% CI (mean±2 s.e.m.) for replacement rate at each pre-exposure treatment.

## RESULTS

### A broad hormetic curve with asymmetric slopes relates survival resilience to pre-exposure temperature

**Fig. 3.**
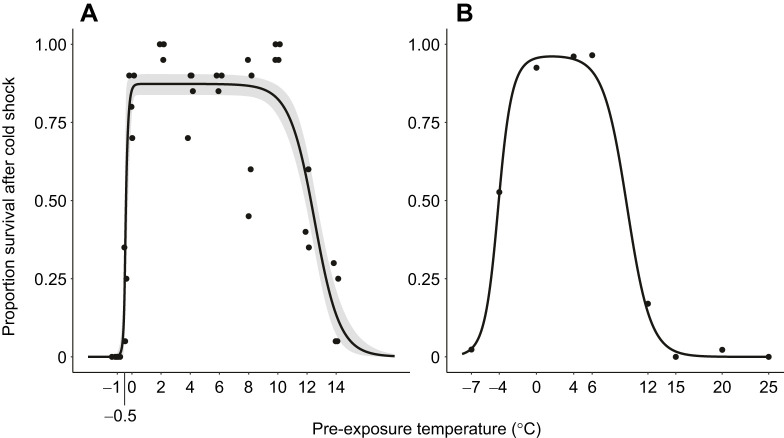
**Survival resilience of *Drosophila melanogaster* and *Sarcophaga crassipalpis*.** (A) Survival resilience (post cold shock) of 4- to 5-day-old female *D. melanogaster* versus pre-exposure temperature. All flies were held at the pre-exposure temperature for 210 min followed by 60 min of stress exposure at −7°C. The black solid line and the gray shaded area are the fitted bi-logistic model and the 95% CI, respectively; filled black circles are the mean proportion survival of each replicate vial, jittered horizontally to limit overlap. (B) Survival resilience of pharate adults of *S. crassipalpis* versus pre-exposure temperature. Filled black circles are mean proportion survival estimated by Chen et al. (1987); the solid black line is the fitted bi-logistic model (CI not estimated because the model was not fitted to raw data).

The bi-logistic model fit the survival data well and suggested a broad range of temperatures over which pre-exposure to moderately low temperature improved survival resilience to cold shock (Fig. 3A, Table 1). Low survival resilience with a pre-exposure of 14°C suggests that higher temperatures are inadequate to stimulate a hardening response. The slope of the increasing phase (survival increases with decreasing temperature) at higher pre-exposure temperatures was relatively steep, with the mean predicted survival increasing from 14% to 83% survival over 4°C from 14°C to 10°C pre-exposure treatments. This translates to a mean *Q*_10_ of 0.13, the equivalent steepness (inverse) of a *Q*_10_ of 7.7 for a rate increasing with temperature over the same range. This pattern is consistent with a relatively steep threshold process wherein decreasing pre-exposure temperature lower than 10°C (and therefore applying a more intense pre-treatment) provides no greater protection from cold-shock-induced chilling injury. Though there was substantial variability (illustrated by the variation in vial means), predicted mean survival resilience remained similarly high from pre-exposures between 10°C and 0°C, quite a broad range of temperatures that induced similar resilience.

**Table 1.** Parameter estimates for the bi-logistic model fit to *Drosophila melanogaster* survival data including the mean and 0.05 and 0.95 quantiles of the posterior distributions

Parameter	Posterior mean	90% CI
*k*_1_	16.3	6.86, 37.0
*k*_2_	−1.17	−1.47, −0.89
*i*_1_	−0.407	−0.475, −0.313
*i*_2_	12.5	12.2, 12.8
*L*	0.873	0.844, 0.900

The parameters *k*_1_ and *k*_2_ model the steepness of logistic change, *i*_1_ and *i*_2_ model the position of the inflection points, and *L* models the maximum survival proportion.

Survival resilience declined precipitously at pre-exposure temperatures below 0°C, dropping from mean predicted survival of 85% to 0% survival from 0°C to −1°C with a mean *Q*_10_ of 2.9×10^34^. This suggests an extremely steep threshold for chilling damage, inhibition of physiological processes that underlie hardening, or some combination of both. This decline is not likely related to internal ice formation, which does not occur until below −16°C in adult *D. melanogaster* flies (Czajka and Lee, 1990). The fitted model predicts survival resilience in this temperature range that is higher than zero (the outcome with no pre-exposure) yet much lower than the survival resilience induced at the plateau of the hormetic curve.

### A similar hormetic curve relates survival resilience to pre-exposure temperature in the flesh fly *Sarcophaga crassipalpis*

Data from Chen et al. (1987) reveal a very similar relationship between pre-exposure temperature and survival resilience in a different life stage (pharate adults) in a different fly species (*S. crassipalpis*). The fitted bi-logistic model suggested clear pre-exposure temperature thresholds bracketing a range of pre-exposure temperatures over which survival resilience was similarly high (Fig. 3B), albeit over a narrower range than for *D. melanogaster* adult females. Estimated mean *Q*_10_ coefficients were 0.078 for the increasing phase (inverse *Q*_10_=12.8) and 216 for the decreasing phase.

### Pre-treated, surviving flies had universally low, but non-zero, fertility

**Fig. 4.**
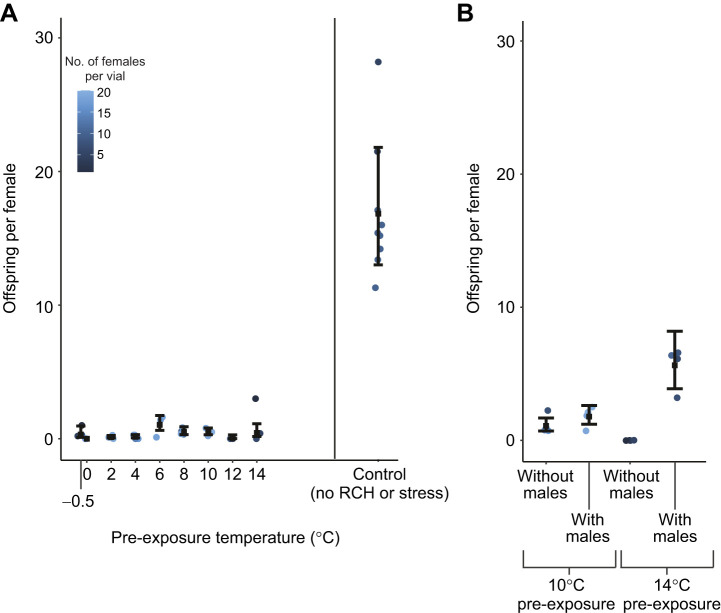
**Effects of pre-exposure temperature and male availability on female fertility.** (A) Post-stress fertility of females without access to males compared with untreated controls. (B) The combined effects of pre-exposure temperature and male addition on fertility. All flies were held at the pre-exposure temperature for 210 min followed by 60 min of stress exposure at −7°C as in the survival experiments. Each filled circle represents offspring per surviving female per replicate vial; post-stress, vials varied in the number of surviving females (blue shading). Black, filled squares represent the mean of each treatment estimated from fitted generalized linear models; black whiskers represent 95% CI of the means. All replicate vials yielded zero offspring for 0°C pre-exposure (A) and 14°C pre-exposure without males (B) – these treatments thus do not have associated 95% CI.

In contrast to survival, fertility resilience was universally low compared with fertility in control flies that did not experience pre-exposure cold or cold shock (Fig. 4A). There was a weak relationship with pre-exposure temperature, with fertility slightly higher from 6°C to 10°C. This pattern hints at a hormetic relationship similar to survival, but the outcome was highly stochastic. For example, fertility was zero in the 0°C treatment despite reasonably high survival, but non-zero in the −0.5°C treatment where survival was low. Addition of unshocked males back to groups of cold-shocked females to allow for post-recovery mating had only marginal effects on fertility resilience, and only in the 14°C pre-treatment (full model, including interaction AIC=102.6; reduced model excluding interaction AIC=126.2) in which fertility resilience was moderately higher with the availability of males compared with no males (Fig. 4B). However, fertility resilience of all flies in pre-exposure plus cold-shock treatments was much lower than fertility of control flies.

**Fig. 5.**
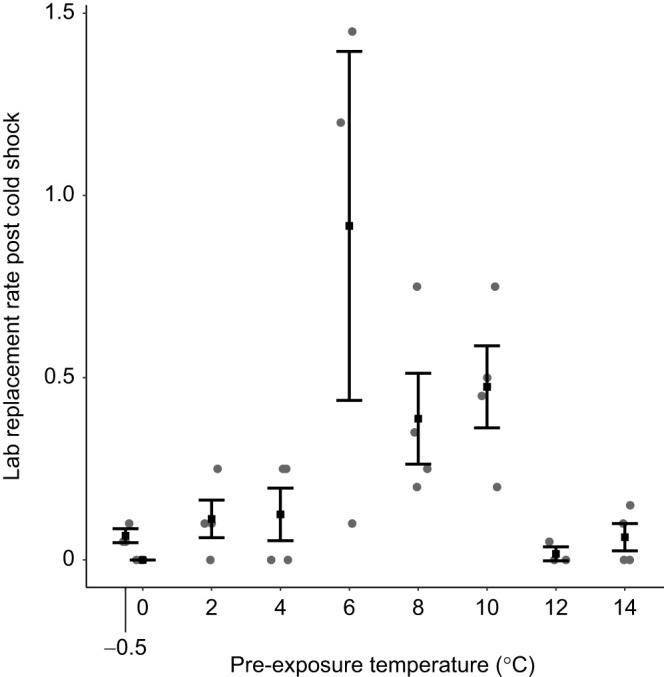
**Replacement rate (survival proportion×offspring per female) estimated for each pre-exposure temperature in which both survival and fertility (pupae per female) were measured (excluding −1°C at which survival was zero; without addition of males post-cold shock).** Each filled gray circle represents a replicate vial, filled black squares represent estimates of the mean, and error bars are approximate 95% CI (mean±2 s.e.m.; except for 0°C pre-exposure, at which no offspring were produced).

Overall, pre-exposure to mildly cold temperatures allowed flies to successfully reproduce following cold shock in the laboratory. Estimated replacement rates (survival×offspring per female) following cold shock under laboratory conditions were low but clearly non-zero at most pre-treatment temperatures that allowed survival (Fig. 5). Laboratory estimates of replacement rate were also highest at intermediate pre-exposure temperatures driven by higher survival resilience and slightly higher estimates of fertility resilience.

## DISCUSSION

### Modeling cold pre-exposure as a hormetic process

The bi-logistic model that we implemented based on conventions for hormetic processes (Beckon et al., 2008) provides a useful quantitative framework to describe what appear to be sharp, threshold relationships between pre-exposure temperature and survival resilience. The model can also be fit to different distributions such as the negative binomial for fertility rates (Supplementary Materials and Methods), though we used GLMs in our analysis of fertility. More flexible models could easily be applied to fit different curve shapes using other forms of the bi-logistic or other biphasic functions (Brain and Cousens, 1989; Hoekstra, 2006; Beckon et al., 2008), providing a flexible framework for understanding how acclimation and injury interact to influence phenotypes as pre-exposure temperatures approach lethal limits.

Here, our hormetic model fit is consistent with relatively steep, threshold processes for beneficial acclimation and cold injury separated by a relatively broad range of temperatures over which pre-exposure induces high survival resilience in *D. melanogaster*. The observation of a broad range of effective pre-exposure temperatures aligns well with the combined results of multiple studies suggesting that RCH (with respect to survival) occurs with sufficiently long pre-exposures (typically >1 h) at 0–5°C in *D. melanogaster* (Chen and Walker, 1994; Yi et al., 2007; Overgaard et al., 2007; Gerken et al., 2015). Moreover, results from Chen et al. (1987) show a very similar plateau-shaped relationship in pharate adult *S. crassipalpis* (Fig. 3B). The cold hardening response seems to be at least partially cell autonomous (Nadeau and Teets, 2020), perhaps explaining the conservation of RCH across insect life stages and species (Teets et al., 2020). Concordance between the results of the present study and Chen et al. (1987) further suggests a broader conservation of the plateau-shaped, hormetic relationship implying temperature thresholds for hardening and cold injury.

### A note on the use of a mathematical definition of resilience

In this study we did not use an explicit mathematical definition of resilience. Instead, we simply report survival and fertility metrics on an absolute scale. Resilience could be mathematically defined as a fold change, i.e. the ratio of the measured fitness response following stress at a given environmental exposure (here, cold pre-exposure) over the fitness response under some control exposure (e.g. pre-exposure at the standard rearing temperature). Resilience values greater than and less than 1 would then suggest hardening or detrimental responses, respectively. This approach could work well in many experimental designs if the control set of conditions yield non-zero values for the measured fitness component. In our study, flies had zero survival when pre-exposed to the standard rearing conditions (24°C). Thus, we would need to use another, arbitrary exposure as the ‘control’ to estimate fold changes for any fitness component. Though we found a specific, mathematical definition of resilience impractical for our study, we support using fold change or similar, quantitative metrics of resilience in studies of environmental hormesis or hardening when possible.

### Limited protection of reproduction by cold pre-exposure

Fertility was low across all tested pre-exposure temperatures that enhanced survival resilience (about 85% lower than control flies). Nevertheless, some females successfully reproduced following cold shock when they were pre-exposed to more moderate cold. However, we note that because fertility was measured at the group level, we cannot determine whether these fertility patterns reflect uniform reductions across all surviving individuals, or heterogeneous responses where some individuals maintained normal fertility while others were rendered sterile. A previous study of cold hardening in *D. melanogaster* likewise showed fertility resilience to cold shock (–5°C for 1 h) with cold pre-exposure (ramp down from 25°C to 0°C at −0.1°C min^−1^ followed by 1 h at 0°C), yet fertility was substantially reduced compared with unstressed controls (Overgaard et al., 2007). In contrast, both the present study and Overgaard et al. (2007) found that RCH maintained survival at relatively high levels compared with unstressed controls, at least under some pre-exposure treatments. A study in *S. crassipalpis* also found more drastic reductions in fertility (∼50% decline in fertilized eggs) compared with survival (Rinehart et al., 2000). There is one report that RCH can produce very high fertility resilience in *D. melanogaster*; pre-exposure to cold yielded post-cold-shock fertility rates indistinguishable from those of unstressed controls (Kelty et al., 1999). However, that study applied a substantially less intense cold shock (−3°C for 1 h, using the same Oregon R strain as the present study) and measured egg production but not viability through pupation. Overall, results from studies in *D. melanogaster* suggest that even when pre-exposures to cold lead to high survival resilience, extreme cold exposures may severely limit reproductive success regardless of temperature pre-conditioning.

### Ecological implications

RCH and similar experiments are often designed to simplify and isolate thermal effects, rather than to emulate ecological reality. However, RCH can occur at ecologically relevant cooling rates that reflect, for example, daily temperature fluctuations in the field (Kelty et al., 1999). Further, insects sampled directly from the field at different times of day also demonstrate cold tolerance consistent with RCH (Kelty, 2007).

Survival of fluctuations from moderate to extreme cold in nature surely reflect the offsetting effects of hardening and cold injury accumulating over time. Cold injury appears to accumulate at a constant rate over time at a given temperature, and that rate is negatively, log-linearly related to temperature in adult *Drosophila suzukii* (Tarapacki et al., 2021). This result is consistent with the thermal death time (TDT) models developed to predict heat tolerance in *D. melanogaster* based on a similar log-linear (positive) relationship between temperature and injury rate (Jørgensen et al., 2019). However, the TDT models predict survival under dynamic exposure to hot temperatures remarkably well under the assumption of no hardening (Jørgensen et al., 2019). In contrast, pre-exposure at moderately low temperatures has a strong, positive effect on survival resilience following a cold shock that initially accumulates over time, then eventually declines at longer exposure durations (Chen and Walker, 1994). Tarapacki et al. (2021) have also shown that long-term, egg-to-adult acclimation shifts the intercept of the log-linear relationship between temperature and cold injury rate.

Modeling the effects of dynamic temperatures on survival in cold temperatures will therefore require integrating the TDT model with models relating temperature to hardening effects. Our hormetic model shows that hardening effects are not log-linearly related to temperature – for example, the beneficial effect of pre-exposure on survival resilience is similar across the entire plateau of the hormesis curve. These results predict that sufficient time spent at any temperatures spanning the hormesis curve plateau should have similar hardening effects. Indeed, different cold ramping rates in RCH experiments can have equivalent effects on survival resilience in *D. melanogaster* even though flies in the different treatments experience different durations of exposure to different temperature ranges (Kelty et al., 1999). A model combining TDT and hardening effects would therefore need to include an additional component accounting for the non-linear relationship between temperature and hardening. Developing such a model would require reasonably large, factorial experiments applying a range of static temperatures and durations of exposure for predictions and dynamic temperature for validation similar to those described in Jørgensen et al. (2019).

Fertility was also maintained across a broad range of pre-exposure temperatures, albeit at relatively low levels. In turn, our laboratory estimates of replacement rate were non-zero under most pre-exposure conditions, and highest within the same range of pre-exposure temperatures that maximized survival. Our experiments tracked fertility only for 48 h, and flies may attain higher reproductive output over longer time scales, though this has only been measured following more moderate cold exposures (El-Saadi et al., 2020; Kelty et al., 1999). Overall, the results suggest that natural populations of flies may therefore persist in the face of severe cold stress in naturally fluctuating environments, provided that replacement rates are also non-zero in nature and eventually recover within or across generations (replacement rates below 1 lead to population decline).

In contrast, fertility may be the primary limiting factor for persistence in environments where bouts of extreme cold are relatively common. Though we did not test colder extreme temperatures (below −7°C), we suspect that there are ecologically plausible combinations of extreme cold and pre-exposure that maintain some fly survival but do not permit enough reproduction for population persistence in nature. Fertility limits at high temperature appear to play an outsized role in predicting geographic ranges and climate change vulnerability (Walsh et al., 2019; van Heerwaarden and Sgrò, 2021; Parratt et al., 2021). However, most studies relating reproductive output at low temperatures to geographic location or range limits have investigated responses to chronic cold rather than short-duration, extreme cold exposure (e.g. Overgaard et al., 2014; Chakir et al., 2002; Vollmer et al., 2004). Evidence for similarity in cold injury owing to long- versus short-term exposure is mixed (Sinclair and Roberts, 2005), though it is well known that long-term cold exposure can induce diapause or quiescent reproductive arrest in many insects, including *D. melanogaster*, protecting reproduction by delaying until conditions become permissive (Mensch et al., 2017; Schmidt et al., 2005). Overall, current evidence from the literature does not provide a clear message about whether and how reproductive resilience to acute, cold-induced stress may influence ecological outcomes that in turn may influence geographic range limits.

### Physiology – accumulation of RCH, then damage

What physiological processes might underlie the upper temperature threshold for RCH that we observed? There is sound evidence that several processes may contribute to RCH. These include adjustments to membrane fluidity, calcium ion concentration and calcium signaling, cryoprotectant accumulation, autophagy, and others that are well summarized in Teets et al. (2020).

Whatever processes underlie the upper RCH threshold, they appear to have a stronger thermal sensitivity (inverse *Q*_10_ of about 7–12) compared with developmental and metabolic processes (*Q*_10_ of about 2–5). Of course, chemical reaction rates are unlikely drivers simply because they are declining with decreasing pre-exposure temperature. Rather, the relatively steep increase in survival resilience with decreasing pre-exposure temperature supports rapid induction of physiological change below some threshold temperature.

To our knowledge, there are no studies that systematically explore the relationship between any candidate RCH mechanisms and temperature – rather, specific physiological processes are generally investigated at one or more time points during static temperature exposures. Studies applying a range of pre-exposure temperatures could provide robust additional evidence for a given candidate mechanism if survival resilience and some indicator metric (e.g. transcript or protein, ion or metabolite abundance) were correlated across pre-exposure temperatures. Such experiments might also reveal whether different sets of physiological processes contribute to hardening at different pre-exposure temperatures.

On the other end of the hormesis curve, we hypothesize that the decline in survival resilience at lower temperatures reflects the accumulation of cold injury. Cold temperatures will eventually inflict damage at low enough temperatures, but this is not sufficient evidence for cold injury at pre-exposure temperatures below 0°C in *D. melanogaster*. It is also possible that the physiological processes contributing to hardening simply become less effective or inhibited at lower temperatures. However, Chen and Walker (1994) show that the hardening effect (increased survival of a cold shock) of pre-exposure to 0°C and 4°C starts to exponentially decline at exposure durations that also cause declining survival without a cold shock. Moreover, the extremely high *Q*_10_ coefficients that we estimated for the decreasing phase of survival resilience (2.9×10^34^) exceeds even the already extreme *Q*_10_ coefficients for survival time decline at stressful high temperatures (*Q*_10_ up to 10,000 or more; Maynard Smith, 1957; Jørgensen et al., 2022). Finally, *Q*_10_ coefficients for the inverse U-shaped survival curves relating egg to adult viability to rearing temperature were also very high (*Q*_10_ from 1×10^3^ to 4×10^6^) relative to those expected for enzymatic and metabolic processes at relatively low temperatures (van der Have, 2002). Models based on deactivation of cell cycle proteins at relatively extreme temperatures are consistent with steep declines at low (and high) temperature observed for egg to adult viability in *Drosophila* and other insects (van der Have, 2002). Though cell cycling per se is unlikely to play a crucial role during the relatively short thermal exposures that we applied to post-metamorphic adults, inactivation of other, critical proteins could contribute to the observed pattern. For example, rapid loss of ion homeostasis is associated with mortality in the cold (MacMillan et al., 2015b), and threshold inactivation of proteins promoting ion balance could produce the precipitous declines in survival resilience that we have observed at low pre-exposure temperatures.

Although somatic damage may be low across a wide range of pre-exposure temperatures that promoted high survival resilience, female reproductive organs and/or stored sperm are either damaged at pre-exposures below 14°C, or hardening processes are not effective at protecting reproductive tissues from cold shock. Exposure to temperatures from 4°C to 11°C can reduce fertility in *D. melanogaster*, but only at durations exceeding 24 h (Mockett and Matsumoto, 2014). Thus, injury to stored sperm or female reproductive tissue seems unlikely at pre-exposures at and above 4°C in our experiment.

Rather, our results suggest that most pre-exposure temperatures that protect against somatic injury do not protect against injury to both stored sperm and female reproductive tissue. As little as 15 min at −5°C can kill all stored sperm in the female spermathecae and seminal receptacle of *D. melanogaster*, though cold-shocked females can lay a few viable eggs that were previously fertilized (Novitski and Rush, 1949). It is therefore likely that our cold shock at −7°C for 1 h killed all stored sperm, and that pre-exposure to moderate cold offered little, if any, protection. Moreover, the few offspring that were successfully produced probably hatched from eggs fertilized prior to cold shock treatment. We thus suggest that cold injury to sperm and/or reproductive tissue contributes to low fertility, though it possible that the treatments also inhibit motivation or ability to successfully mate. Novitski and Rush (1949) also note that, though stored sperm is killed during cold shock at −5°C, female fertility rebounds when they are mated to fertile males. We observed a minimal fertility rebound in females with access to males and with a pre-exposure to 10°C, and a higher, moderate rebound with a pre-exposure of 14°C, though both had substantially lower fertility compared with untreated controls. This suggests some damage to unfertilized eggs and/or reproductive tissues that persists at least 48 h after cold shock. It is possible that damage is repaired and fertility rebounds to higher levels with more recovery time; other studies suggest fertility resilience when females are allowed to mate for five or more days following cold shock (Novitski and Rush, 1949; Kelty et al., 1999). Nevertheless, our results clearly demonstrate that pre-exposures that protect against mortality do not necessarily protect reproductive output.

### Conclusions

Our data show a hormetic curve with a broad range of pre-exposure temperatures that effectively improve cold shock survival but yield universally low fertility. The fit of our biphasic model to these data suggests threshold processes underlying both RCH and cold injury, with little overlap in temperature range (cold injury does not seem to accumulate until well past the threshold for RCH). Further investigation associating hormetic curves among traits such as survival and candidate physiological processes may help elucidate the mechanisms underlying RCH and cold injury.

## Supplementary Material

10.1242/jexbio.250856_sup1 Supplementary information
