# Supplementary material for: When is RCH not RCH? Rapid cold hardening has steep temperature thresholds inducing high survival but low fertility resilience to cold stress
Source: J Exp Biol. 2026 Jan 30;229(2):jeb250856. doi: 10.1242/jeb.250856 (PMC12891942; doi:10.1242/jeb.250856)
Supplement: Supplementary information [file jexbio-229-250856-s1.pdf]

Supplementary Materials and Methods

Bayesian model fitting

I. Survival

*Note that the table of estimated parameter values and a plot of the fitted function appear in the main text.

The generic, bilogistic function that we used to model hormesis is:

$$R = \frac{L}{1 + e^{-k_1(t-i_1)} + e^{-k_2(t-i_2)}}$$

where R is a quantity directly measured or calculated from empirical data, t is the temperature, L models the upper limit of R, k_1 and k_2 model the steepness of logistic change in the decreasing and increasing phases, respectively, and i_1 and i_2 model the temperatures of the two inflection points. For survival we modeled the survival proportion ($R = \theta_s$), with the range [0,1]. This requires that L is constrained to [0,1], though we tightened that constraint in addition to adding other biologically informed constraints and priors. Other details appear in the main text. Constraints/priors were necessary because HMC chain convergence was poor with unconstrained models, presumably because there is some covariation among parameter values. We chose constraints and priors carefully to reflect plausible beliefs about the modeled biological processes.

We used the following constraints:

1) $0.5 < L < 1$: the tighter constraint on L reflects our observation that intermediate temperature treatments consistently yielded mean proportion survival well above 0.8.
2) $-3 < i_1 < 10$: we noted a clear drop-off in survival below 0°C pre-exposure – a drop-off at lower temperatures is inevitable because at some point temperatures drop into a stressful, then non-survivable range. We used lower and upper constraints at relatively higher and lower values that were highly implausible to limit influence on the posterior.
3) $-2 < i_2 < 18$: we noted a clear drop-off in survival above 10°C – a drop-off at higher temperature is also inevitable because an abundance of previous research and our own pilot experiments show that without colder pre-treatments survival after cold shock is very low (zero in our pilots). We used lower and upper constraints at relatively higher and lower values that were highly implausible to limit influence on the posterior.

And, we used the following priors:

1) $k_1 \sim normal(10,20)$: the slope of the relationship was clearly positive for lower values of temperature t (see rationale for constraint 1 above). We used a relatively large standard deviation to reduce prior influence.

Journal of Experimental Biology • Supplementary information

2) $k_2 \sim normal(-10,20)$: the slope of the relationship was clearly negative for higher values of temperature t (see rationale for constraint 2 above). We used a relatively large standard deviation to reduce prior influence.

All other priors were (default) uniform, non-informative, and we used (default) starting values in the interval (−2,2) (see Stan manual).

With larger amounts of data and/or carefully chosen starting values constraints and moderately informative priors might not be necessary for HMC convergence. The purpose of this study was to demonstrate the utility of the function for modeling hormetic responses. Our immediate goals did not include developing and thoroughly testing the generality of the 'best' and/or most practical function, though the parameters of the bilogistic function have easy biological interpretations and the function will fit data from different distributions (see below).

II. Fertility

We also fit the above bilogistic function to fertility rate data. As noted in the main text, the fit of the model was relatively poor and required tight constraints and overly informative priors that likely biased the fit beyond a reasonably broad set of biologically informed 'guesses'. Here we provide the model details and visualization of the fit to demonstrate the generality of the bilogistic model to alternative data distributions.

We modeled fertility rate ($R = f$), calculated as the number of pupae produced in a vial divided by the number of surviving females in a vial. Thus, we modified the bilogistic as follows to use the number of surviving females N as an offset:

$$f = \left(\frac{L}{1 + e^{-k_1(t-i_1)} + e^{-k_2(t-i_2)}}\right) N$$

We used a negative binomial likelihood function with the μ,ϕ parameterization, where μ is the mean, and ϕ is the overdispersion parameter (see https://mc-stan.org/docs/functions-reference/unbounded_discrete_distributions.html#nbalt, neg_binomial_2 function). Here, the likelihood function used $\mu = f$, and ϕ was estimated as an additional parameter.

Initial attempts at fitting yielded poor results with poor mixing and convergence using constraints and priors comparably loose to those that we used for the survival model. This is likely because there was high variance and relatively low sample sizes (relatively few replicate vials and low numbers of surviving females).

However, we were able to fit a model that converged using the following constraints and priors:

Constraints:

1) $1 < L < 50$
2) $-3 < i_1 < 18$
3) $-2 < i_2 < 18$

Journal of Experimental Biology • Supplementary information

4) $0 < k_1$
5) $0 < k_2$
6) $0 < \phi < 1000$

Priors:

1) $k_1 \sim normal(2,2)$
2) $k_2 \sim normal(-2,2)$
3) $c_1 \sim normal(4,2)$
4) $c_2 \sim normal(8,2)$

Figure S1 below illustrates the model fit.

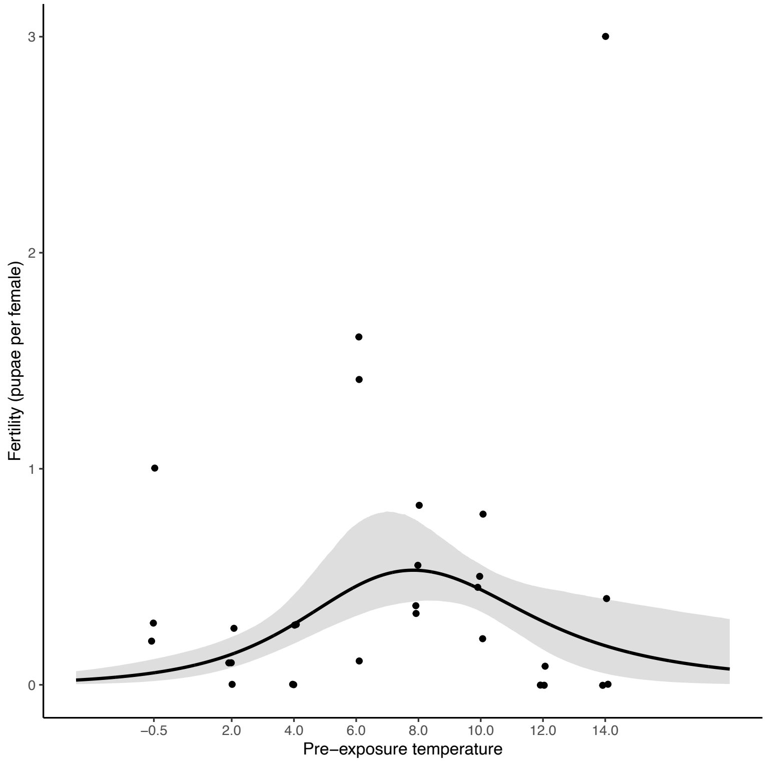

Fig. S1. Bayesian model fit to fertility data. Each filled circle represents a replicate vial, the solid line is the model predicted mean, and the grey ribbon is the 90% credible interval.

Table S1. Drosophila diet recipe

# of vials	Cornmeal (g)	Yeast (g)	Agar (g)	DI H2O (BOIL) (mL)	DI H2O (Mix) (mL)	Tegosept (g)	Molasses (mL)	Propionic Acid (mL)	Ethanol (mL)
100	64.5	13	6.7	0.6	0.3	1	65	5.4	4
200	129	26	13	1.2	0.6	1	130	11	8
300	193.5	39	20	1.8	0.9	2	195	16	12
400	258	52	27	2.4	1.2	3	260	22	16
500	322.5	65	34	3	1.5	4	325	27	20
